# Neural Temporal Dynamics of Social Exclusion Elicited by Averted Gaze: An Event-Related Potentials Study

**DOI:** 10.3389/fnbeh.2018.00021

**Published:** 2018-02-06

**Authors:** Yue Leng, Yanmei Zhu, Sheng Ge, Xing Qian, Jili Zhang

**Affiliations:** ^1^School of Biomedical Science and Medical Engineering, Southeast University, Nanjing, China; ^2^Research Center for Learning Science, Southeast University, Nanjing, China; ^3^Key Laboratory of Child Development and Learning Science (Ministry of Education), Southeast University, Nanjing, China; ^4^Institute of Child Development and Education, Southeast University, Nanjing, China

**Keywords:** social exclusion, eye gaze, event-related potentials, P200, LPP

## Abstract

Eye gaze plays a fundamental role in social communication. The averted eye gaze during social interaction, as the most common form of silent treatment, conveys a signal of social exclusion. In the present study, we examined the time course of brain response to social exclusion by using a modified version of Eye-gaze paradigm. The event-related potentials (ERPs) data and the subjective rating data showed that the frontocentral P200 was positively correlated with negative mood of excluded events, whereas, the centroparietal late positive potential (LPP) was positively correlated with the perceived ostracism intensity. Both the P200 and LPP were more positive-going for excluded events than for included events. These findings suggest that brain responses sensitive to social exclusion can be divided into the early affective processing stage, linking to the early pre-cognitive warning system; and the late higher-order processes stage, demanding attentional resources for elaborate stimuli evaluation and categorization generally not under specific situation.

## Introduction

In social life, everyone may experience exclusion from social relationship or social interaction, such as falling out of love, break-up with friends. Social exclusion or ostracism may lower basic need satisfaction including belonging, control, meaningful existence, self-esteem (Wirth et al., 2010), decrease prosocial behavior (Twenge et al., 2007; Moor et al., 2012), increase degrees of internalizing problems such as anxiety and depression (Deater-Deckard, 2001; Ladd, 2006) and even lead to antisocial behavior, like aggression (Twenge et al., 2001).

Due to the social nature of human beings and the essential need of social affiliation, social exclusion is often accompanied with social pain. Considerable neuroimaging studies have reported that experience of social exclusion elicited by ball-tossing game (Eisenberger et al., 2003; Masten et al., 2009; DeWall et al., 2010; Bolling et al., 2011; Moor et al., 2012), negative social evaluation which involves receiving rejecting feedback from others (Eisenberger et al., 2011), and even the cues that represent social rejection (Kross et al., 2007), can activate affective physical pain-related neural regions including dorsal anterior cingulate cortex (dACC) and anterior insula (AI). Moreover, the activities of these brain regions often appeared to be positively correlated with self-report social distress (Eisenberger et al., 2003; Krill and Platek, 2009; Onoda et al., 2009; DeWall et al., 2012). In addition to the dACC and AI, some other activated neural regions in response to excluded events relative to included events were also been reported. For example, for the adult, the increased activities of right ventral prefrontal cortex (Eisenberger et al., 2003; Masten et al., 2009), hippocampi, left ventrolateral prefrontal cortex and left middle temporal gyrus (Bolling et al., 2011) were reported following social exclusion than following social acceptance. For the adolescent, subgenual ACC and ventral striatum activated significantly in response to social exclusion (Masten et al., 2009; Moor et al., 2012). However, the cortical regions including superior temporal gyrus cortex, insula, ACC and subcortical regions including striatum, thalamus activated greatly after acceptance than after rejection feedback (Guyer et al., 2012).

However, the temporal dynamics of neural responses to social exclusion is still debated. In recent years, several event-related potential (ERP) studies have shed light on it (Crowley et al., 2009, 2010; Gutz et al., 2011; van der Veen et al., 2014). Some ERP components associated with social exclusion have been reported successively. Firstly, the slow-wave activity (occurring between 400 ms and 900 ms post-stimulus) in the left prefrontal/medial frontal cortical regions of young adults was negatively associated with self-report distress during both complete-rejection and micro-rejection events (Crowley et al., 2009). In their subsequent study, social exclusion also evoked more negative slow wave in the medial frontal regions of adolescents (Crowley et al., 2010). These two studies suggested in agreement that the frontal slow wave might be linked to cognitive regulation of negative emotion induced by social exclusion. In addition, Crowley et al. (2010) also found that, compared to the inclusion events, the exclusion events even elicited more positive P300 and late positive potential (LPP) over posterior regions. The authors suggested that these two ERP components might index the processing of attentional distribution, due to salience of the exclusion events. Subsequently, Gutz et al. (2011) further distinguished between an early fronto-central P3a and a late parietal P3b elicited by social exclusion. It is widely believed that the P3a reflects a “stimulus-driven” frontal exogenous attention mechanism during task processing, whereas, the P3b reflects a parietal Endogenous attention mechanism (Polich, 2007; Kaunhoven and Dorjee, 2017). With the addition of the correlation between ERP components amplitudes and subjective-ratings scores, in the context of social exclusion paradigms, it has been suggested that the P3a may be associated with the affective processing of exclusion (Gutz et al., 2011; van der Veen et al., 2014), whereas, the P3b may be related to its perceived excluded intensity (Gutz et al., 2011).

Up to now, Ball-tossing (or Cyberball; Eisenberger et al., 2003; Masten et al., 2012; Moor et al., 2012) and Chatroom Task (or Chatroom Interact Task; Guyer et al., 2008, 2009, 2012; Silk et al., 2012) have been considered as two major experimental tasks used to investigate neural and physiological basis of social exclusion. As many researchers have proved, Cyberball is an efficient paradigm to explore children and adolescents’ neural processes of social exclusion and social pain. However, it seems less appealing to adults leading to lower ecological validity. In addition, the modified Cyberball in ERP experiments makes participants feel whether they are accepted or excluded by the other partners after a period of time, as a result, some ERP components related to the rapid neural responses may not be detected. Recently, the emergence of some novel paradigms may provide new opportunities to overcome these deficits. For example, Wirth et al. (2010) developed an Eye-gaze paradigm for a behavioral study of social ostracism, during which the participants were asked to imagine interacting with an individual on the computer screen displaying averted or direct eye gaze. In that study, the thwarted basic need satisfaction, reduced explicit and implicit self-esteem, lowered relational value, and increased temptations to act aggressively toward the interaction partner suggested that the averted eye gaze is a nonverbal cue to indicate a form of ostracism. Importantly, the direction of eye gaze may deliver the feedback signal (i.e., exclusion or inclusion) more straightforwardly and immediately.

Therefore, some general earlier ERP components related to affective processing may also be involved in the time course of social exclusion. By using the affective pictures stimuli, many studies have found that the P200 peaking at the latency of 200–300 ms at fronto-central area was significantly greater for the negative picture stimuli than for the positive picture stimuli, reflecting attentional negativity bias involved in the early stage of emotion perception (Carretié et al., 2001; Huang and Luo, 2006; Muñoz and Martín-Loeches, 2015). In some studies, the P200/N200 complex is regarded as an index of early attention, with stronger responses to negative emotional stimuli than to positive stimuli (Feng et al., 2014).

The model of ostracism was proposed first in 1997 (Williams, 1997) and revised subsequently (Williams and Zadro, 2005). Based on abundant results of the behavioral and neuroimaging research, the latest temporal need-threat model of ostracism proposed by Williams describes and predicts processes and responses at three stages of reactions to ostracism, i.e., the reflexive, the reflective and the resignation. During the reflexive stage, ostracism is felt as pain and as a threat to four fundamental needs: belonging, self-esteem, control and meaningful existence. This stage often last for a few seconds. During the reflective stage, ostracized individuals reflect on the meaning and relevance of the ostracisms experience, leading to coping responses. During the resignation stage, persistent exposure to ostracism over time depletes the resources necessary to motivate the individual to fortify threatened needs, thus leading eventually to resignation, alienation, helplessness and depression (Williams, 2009; Williams and Nida, 2011).

Therefore, to detect the temporal dynamics of brain responses to social exclusion, the current study employed a modified Eye-gaze paradigm in a real-life scenario—University club recruitment. The stimulus pictures presented in the ERP experiment were the front-view faces of ten interviewers who attended the interview. The interviewers’ direct or averted eye gaze towards the interviewees may deliver the signal whether they inclined to accept or reject the interviewees straightly. Based on the temporal model of ostracism and previous research on the time course of social exclusion and emotional processing, we predicted that, first, the late slow wave associated with controlled cognitive processing would response greatly to the excluded events compared to the accepted events. Second, since the signal of being excluded or included conveyed by the direction of eye gaze can be perceived rapidly, we hypothesized some early ERP components related to rapid affective response and stimulus detection would also exhibit differences between the averted and direct gaze.

## Materials and Methods

### Participants

Fifteen freshmen (9 females, age range 18–21 years) in Southeast University who attended University club recruitment participated undergoing the EEG test in the study. They were right-handed, had no history of neurological problems and had normal or corrected-to-normal vision. Ten undergraduate students (5 females, age range 18–21 years) who were strangers to all the EEG participants were recruited as interviewers. The EEG participants and interviewers were paid 30 RMB and 10 RMB for their participation, respectively, and given informed consent, which was approved by the Ethics Committee of Affiliated Zhongda Hospital, Southeast University, China (2016ZDSYLL002.0).

### Apparatus and Procedure

The task consisted of two sessions. The first session is the University club recruitment interview. Each freshman signed up the University club freely, and it was the first time for each freshman to be interviewed for joining the club. Ten interviewers were introduced to the interviewee one by one. All the interviewees were informed that whether they got admitted to the club depended on all interviewers’ decisions. In 2 days following the interview, but prior to the admission results being announced, 15 interviewees were invited as the participants to the EEG laboratory to complete the ERP session. All the participants were told that we had snapshot many front-view face pictures of ten interviewers at different time points during the interview, they would report their feelings after these stimulus pictures presented on the computer screen. In fact, unknown to the participants, those pictures had been taken and standardized before the interviewers acted in the club interview. For each interviewer, we obtained four different types of front-view face pictures, i.e., the direct gaze, the closed-eyes, the left-averted gaze and the right-averted gaze.

The EEG participant was seated about 57 cm in front of a 14-inch LCD monitor (screen resolution: 1024 × 768, refresh rate: 85 Hz, color quality: highest 32 bit) in a dimly lit sound-attenuated room undertaking the EEG test. Each trial began with the presentation of a fixation sign (a small white cross subtended 0.7° × 0.7° of visual angle) in the center against black background. After 500 ms, a direct gaze picture presented. After 1000 ms, a closed-eyes picture presented for a random duration of 800–1000 ms. Then, the target stimulus appeared: a direct gaze picture or an averted gaze picture (i.e., either left- or right-averted). After 1500 ms, the participants was asked to press button to select how much they feel distress (i.e., negative mood) and being excluded (i.e., perceived ostracism intensity) ranging from “not at all” to “very much” (1–4 points), respectively. The sequence of the direct gaze and averted gaze picture was determined by a pre-specified pseudorandom sequence, with half the times direct gaze and another averted gaze.

The ERP experiment task was administered on an Intel Core i3 computer with E-prime 2.0 to control the presentation and timing of stimuli. The formal experiment consisted of 2 blocks of 40 trials each. Each block had 20 trials for the direct gaze, 10 trials for the left-averted gaze and 10 trials for the right-averted gaze conditions, respectively. A practice block containing eight trials was administered before the formal test.

### EEG Recording and Analysis

EEGs were recorded from 64 scalp sites using tin electrodes mounted in an elastic cap (NeuroScan Inc., Herndon, VA, USA) according to the international 10-20 system, with the reference on the left mastoid. Eye blinks were monitored with electrodes located above and below the right eye. The horizontal electro-oculogram (EOG) was recorded from electrodes placed 1.5 cm lateral to the left and right external canthi. All electrode impedances were maintained below 5 kΩ. The EEG and EOG were amplified with a band pass of 0.05–70 Hz and continuously sampled at 500 Hz for offline analysis.

Separate EEG epochs of 800 ms (with 200 ms pre-stimulus baseline) were extracted off-line, time-locked to the onset of target stimuli. Epochs were re-referenced to the linked mastoid electrodes. Ocular artifacts were corrected with an eye movement correction algorithm (Semlitsch et al., 1986). Epochs were baseline-corrected by subtracting from each sample the average activity of that channel during the baseline period. All trials in which EEG voltages exceeded a threshold of ±90 μV during recording were excluded from further analysis. The EEG data were low-pass filtered below 30 Hz.

**Figure 1:**
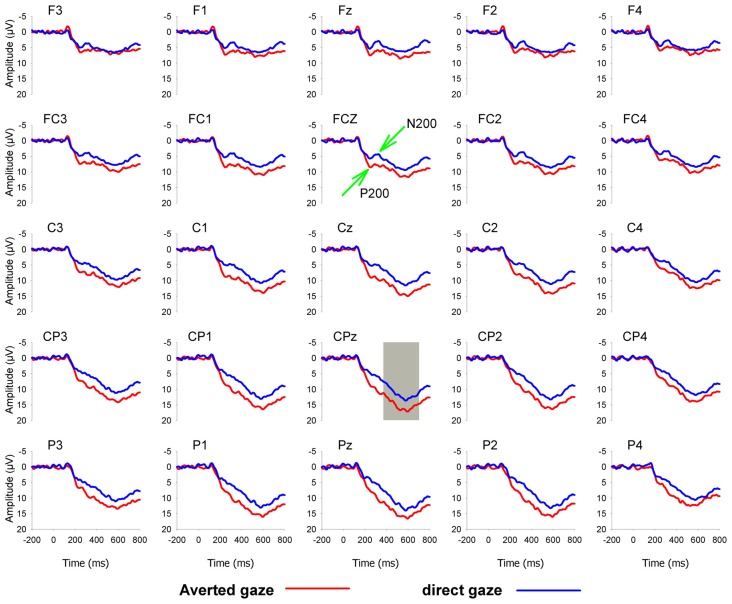
Grand-averaged event-related potential (ERP) waveforms for averted (red lines) and direct (blue lines) eye-gaze pictures at 25 electrodes.

Based on visual inspection of grand-averaged waveforms and scalp topographies, the peak values of the P200 and N200 were detected in the time windows of 170–270 ms and 270–370 ms, respectively, and the mean amplitudes of the LPP were measured in the time window of 370–700 ms. For the purpose of statistical analysis, we focused on 10 frontal, frontocentral and central electrodes, F3, F1, Fz, F2, F4, FC3, FC1, FCz, FC2 and FC4, where the P200 and N200 were the greatest; 25 frontal, frontal-central, central, central-parietal, and parietal electrodes, F3, F1, Fz, F2, F4, FC3, FC1, FCz, FC2, FC4, C3, C1, Cz, C2, C4, CP3, CP1, CPz, CP2, CP4, P3, P1, Pz, P2 and P4, where the LPP was the greatest. Grand average ERP waveforms were shown in Figure 1.

In this study, the ERPs data were cast into a repeated-measures analysis of variance (ANOVA) with three factors for each component: gaze direction (direct vs. averted) and two electrode position factors (laterality and row). The Greenhouse-Geisser correction for violation of the ANOVA assumption of sphericity was applied where appropriate. The Bonferroni correction was used for multiple comparisons.

## Results

### Subjective-rating Data

**Figure 2:**
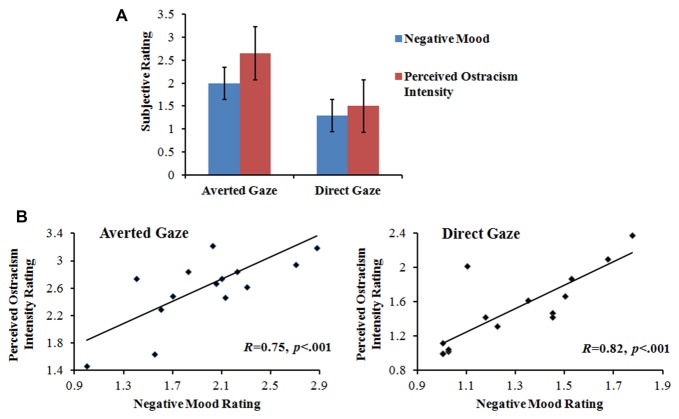
Subjective-rating results. **(A)** Mean subjective rating of negative mood and perceived ostracism intensity for averted and direct gaze conditions. **(B)** The correlation between negative mood rating and perceived ostracism intensity rating for averted and directed gaze conditions.

Repeated-measures ANOVA was conducted separately for the subjective ratings of the feelings of distress (i.e., negative mood) and being excluded (i.e., perceived ostracism intensity), with gaze direction (direct vs. averted) as a within-participant factor. Then, we performed Pearson’s correlation to detect the relationship between those two feelings. Compared to the direct gaze condition, participants in the averted gaze condition felt significantly more painful, *F*_(1,14)_ = 28.97, *p* < 0.001, *η*^2^ = 0.67, and more excluded, *F*_(1,14)_ = 66.68, *p* < 0.001, *η*^2^ = 0.83. In addition, the negative mood was positively correlated to the perceived ostracism intensity on both direct gaze, *R* = 0.82, *p* < 0.001, and averted gaze conditions, *R* = 0.75, *p* < 0.001, as illustrated in Figure 2.

### Event-Related Potentials Data

#### The P200

The three-way repeated-measures ANOVA on the peak amplitudes of the P200 over gaze direction (direct vs. averted), row (frontal vs. frontocentral) and laterality (left, left-central, central, right-central and right; see “Materials and Methods” section) revealed a significant main effect of gaze direction, *F*_(1,14)_ = 9.18, *p* < 0.05, *η*^2^ = 0.40, with the ERP responses being more positive-going to the averted gaze (8.63 μV) than to the direct gaze (6.47 μV). The main effect of row reached significance marginally, *F*_(1,14)_ = 3.46, *p* = 0.084, *η*^2^ = 0.20, with the ERP responses being more positive at the frontocentral area (7.73 μV) than the frontal area (7.37 μV). The main effect of laterality also reached significance, *F*_(4,56)_ = 9.24, *p* < 0.001, *η*^2^ = 0.40, with the ERP responses being more positive at the midline and left-central locations than the other lateral locations. The interaction between gaze direction and row reached significance, *F*_(1,14)_ = 17.23, *p* < 0.05, *η*^2^ = 0.55. The simple-effect analysis showed that for each electrode row, the main effect of gaze direction was significant, *p* < 0.05, with the ERP responses being more positive to the averted gaze than to the direct, and such effect upon direct and averted gaze reached the maximum in the frontocentral area. Only for the averted gaze condition, the main effect of row was significant, *F*_(1,14)_ = 34.30, *p* < 0.001, indicating that averted gaze elicited significantly more positive ERP responses at the frontocentral regions than at the frontal region. However, for the direct gaze condition, the main effect of row did not reach significance, *p* > 0.1. The interaction between gaze direction and laterality also reached significance, *F*_(4,56)_ = 4.21, *p* < 0.05, *η*^2^ = 0.23. The simple-effect analysis showed that for each lateral location, the main effect of gaze direction was significant, *p* < 0.05, with the ERP responses being more positive-going to the averted gaze than to the direct gaze. For the direct gaze condition, the main effect of laterality was significant, *F*_(4,56)_ = 4.14, *p* < 0.05, with the ERP responses being more positive at the midline and right-central locations than at the other lateral locations; and for the averted gaze condition, the main effect of laterality was also significant, *F*_(4,60)_ = 11.28, *p* < 0.001, with the ERP responses being more positive at the midline locations than at any other lateral locations, *p* < 0.05.

The three-way repeated-measures ANOVA on the peak latencies of the P200 over gaze direction (direct vs. averted), row (frontal vs. frontocentral) and laterality (left, left-central, central, right-central and right) did not show neither the main effects nor the interactions between factors.

#### The N200

**Figure 3:**
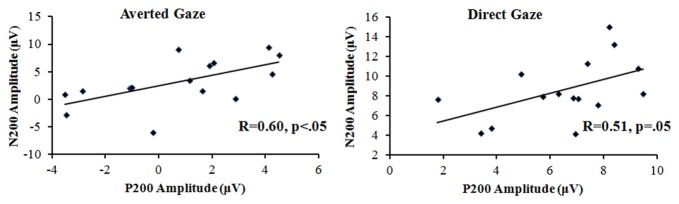
The P200–N200 amplitude correlation for both the averted and direct gaze conditions.

The three-way repeated-measures ANOVA on the peak amplitudes of the N200 over gaze direction (direct vs. averted), row (frontal vs. frontocentral) and laterality (left, left-central, central, right-central and right) revealed a significant main effect of gaze direction, *F*_(1,14)_ = 7.13, *p* < 0.05, *η*^2^ = 0.33, with the ERP responses being more negative to the direct gaze (0.73 μV) than to the averted gaze (3.12 μV). The main effect of row was significant, *F*_(1,14)_ = 20.67, *p* < 0.001, *η*^2^ = 0.60, with the ERP responses being more negative at the frontal area (1.33 μV) than at the frontocentral area (2.52 μV). The main effect of laterality also reached significance, *F*_(4,56)_ = 2.81, *p* < 0.05, *η*^2^ = 0.17, with the ERP responses being more negative-going at right side than any other locations. The interaction between gaze direction and row was significant, *F*_(1,14)_ = 7.93, *p* < 0.05, *η*^2^ = 0.36. The further simple-effect analysis showed that for both direct and averted gaze conditions, the main effect of row reached significance, *p* < 0.05, with the ERP responses being more negative-going at the frontal area than at the frontocentral area. And for each row, the main effect of gaze direction reached significance, *p* < 0.05, with the ERP responses being more negative to the direct gaze than to the averted gaze. In addition, the Pearson’s correlations demonstrated that the positive correlation between P200 and N200 amplitude reached significance highly for the averted gaze condition, *R* = 0.60, *p* < 0.05; and slightly for the gaze condition, *R* = 0.51, *p* = 0.05, which can be shown in Figure 3.

The three-way repeated-measures ANOVA on the peak latencies of the N200 over gaze direction (direct vs. averted), row (frontal vs. frontocentral) and laterality (left, left-central, central, right-central and right) did not show neither the main effects nor the interactions between factors.

#### The LPP

The three-way repeated-measures ANOVA on the mean amplitudes of the LPP over gaze direction (direct vs. averted), row (frontal, frontocentral, central, centroparietal and parietal) and laterality (left, left-central, central, right-central and right) revealed significant main effects of gaze direction was significant, *F*_(1,14)_ = 7.59, *p* < 0.05, *η*^2^ = 0.34, with the ERP responses being more positive-going to the averted gaze (10.91 μV) than to the direct gaze (8.72 μV). The main effect of row, *F*_(4,56)_ = 39.43, *p* < 0.001, *η*^2^ = 0.72, with the ERP responses increasing gradually from frontal to central area, being maximum at centroparietal area, and then decreasing slightly at parietal area. The main effect of laterality also reached significance, *F*_(4,56)_ = 12.10, *p* < 0.001, *η*^2^ = 0.45, with the ERP responses being more positive at the midline location than at any other locations. Furthermore, gaze direction interacted with either row or laterality significantly, *F*_(4,56)_ = 8.73, *p* < 0.001, *η*^2^ = 0.37; *F*_(4,56)_ = 6.07, *p* < 0.001, *η*^2^ = 0.29. The simple-effect analysis revealed that the main effect of gaze direction reached significance at frontocentral, central, centroparietal and parietal areas, *p* < 0.05. For both direct and averted conditions, the main effect of row reached significance, *p* < 0.001, with the ERP responses increasing gradually from frontal to central area, being maximum at centroparietal area, and then decreasing slightly at parietal area.

### Event-Related Potentials and Subjective-rating Data^1^

To explore the effects of exclusion on ERP components and on self-reported data (i.e., negative mood and perceived ostracism intensity), we computed Pearson’s correlations to test the parallel effect. The P200 amplitude of difference waves upon averted minus direct recorded at the left frontocentral area was positively related to the change in negative mood which was calculated as difference between rating for averted gaze and direct gaze, *R* = 0.61, *p* < 0.05. The LPP amplitude recorded at the right parietal area in the averted condition was positively related to perceived ostracism intensity, *R* = 0.53, *p* < 0.05. For the N200 amplitudes, no significant correlation with the self-report data was observed (all *p* > 0.10).

## Discussion

The purpose of the present study is to explore the psychological and ERP responses associated with social exclusion by using the modified Eye-gaze paradigm. The subjective ratings results showed that, compared to the direct gaze condition, participants in the averted gaze condition felt more painful and more excluded. In addition, the distress experience was positively correlated to the feelings of ostracism in both the direct and averted gaze conditions. It has been the first time to employ the Eye-gaze paradigm to investigate individuals’ brain responses to social exclusion, therefore, in contrast to the previous ERP research on social exclusion using other commonly used experimental tasks, like Cyberball, we even found an earlier ERP component related to affective processing (i.e., frontocentral P200), in addition to the late ERP component (i.e., centroparietal LPP) associated with high-level cognitive processing, like elaborate cognitive evaluation and categorization on excluded and included events, and even the attentional allocation to excluded events. The overall brain potentials were more positive-going after the averted gaze than after the direct gaze. In the following paragraphs, we discuss the implication of these findings in detail.

Our findings of subjective ratings preliminarily demonstrate that using the modified Eye-gaze paradigm can investigate the psychological effect of social exclusion effectively and successfully. Previous behavior studies have suggested that compared with the direct gaze, the averted gaze may elicit individuals’ negative emotional response (Schmitz et al., 2012), or lead to avoidance behavior (Itier and Batty, 2009). Specifically, during social interaction, the averted gaze may deliver the signal of negative relational evaluation and even social exclusion (Williams et al., 1998). To improve the ecological validity of the Eye-gaze paradigm developed by Wirth et al. (2010), we modified such the paradigm in the context of University club recruitment interview. Instead of asking the participant to mentally visualize the experience of interacting with the individual whose face was computerized during the movie in the original Eye-gaze paradigm, the participant (i.e., the interviewee) and the individual whose face was computerized (i.e., the interviewer) in our modified paradigm had a real face-to-face interaction during the University club recruitment interview in the early time. Parallel to the previous research on social exclusion, participants in the present experiment generally reported that they felt more painful and more excluded after the interviewers’ averted eye gaze than their direct gaze.

By using emotional stimuli including emotional words (Ponz et al., 2014), emotional pictures (Carretié et al., 2001; Delplanque et al., 2004; Huang and Luo, 2006; Zhu et al., 2015), and even esthetic images (Wang et al., 2012; Muñoz and Martín-Loeches, 2015), numerous affective ERP studies have demonstrated that the frontal P200 amplitudes elicited by negative stimuli are significantly greater than those elicited by positive stimuli. Generally, the P200 component has been linked to early attention according to affective features of the stimuli to be attended. Besides, some other ERP studies presented that frontocentral P3a reflects a “stimulus-driven” frontal automatic attention mechanism during task processing (Sylvain Delplanque et al., 2005; Thierry and Roberts, 2007). Specifically, a few ERP studies on social exclusion have consistently suggested that the frontocentral P3a may be associated with the affective processing of exclusion (Gutz et al., 2011; van der Veen et al., 2014), because of significant positive correlation between the P3a amplitude and subjective-rating scores of negative mood elicited by excluded event. As illustrated in the results of subjective ratings above, the interviewers’ averted gaze in relative to their direct gaze captured during the recruitment interview session, indeed evokes the interviewee’s negative mood (i.e., more painful feeling of exclusion). Moreover, the significantly positive correlation between the change in negative mood and the P200 amplitude of difference waves (i.e., averted gaze minus direct gaze) at left frontocentral area reveals that the frontocenral P200 may be associated with early affective processing of social exclusion. In contrast, such early ERP component was not found in neither Gutz et al.’s (2011) study nor van der Veen et al.’s (2014) study employing Cyberball task which cannot deliver the inclusion or exclusion signal to the participants after each trial. Thus, it can be inferred that the earlier ERP component (i.e., the frontocentral P200) reflecting the affective processing of social exclusion was found in the present experiment, mainly due to the immediacy and high ecological validity of the modified Eye-gaze paradigm we used.

With respect to the processing of affective picture stimuli, an earlier study reported that, in addition to the P200, even the N200 peaking at 240 ms was sensitive to stimulus emotion valence, with more negative-going amplitude for the pleasant stimuli than for the unpleasant stimuli. The authors suggest that such negative deflection should be localized at ventral ACC reflecting the later and deeper processing of automatic attention to emotional stimuli (Carretié et al., 2004). In another study which explored the temporal dynamics of brain activities in the processing of angry, happy and neutral facial expression in a passive viewing task, Schutter et al. (2004) found that compared with the angry facial expression, the amplitudes of anterior P200–N300 complex reduced significantly for the happy facial expression. Importantly, the amplitudes of these two ERP components were positively correlated. The authors proposed that the lowered P200 reflected the initial detection of threat, and the reduced N300 indicated elaborate evaluation of the relevant stimuli (Schutter et al., 2004). In our experiment, the averted eye gaze from the interviewers, which signals ignorance or exclusion, may be perceived as a potential threatening stimulus. Similarly, we also found the P200 amplitudes were positively correlated with the N200 amplitudes for both the averted and direct gaze condition. Therefore, we speculate that the P200/N200 complex induced by the interviewers’ averted gaze in our experiment may reflect the early rapid affective and attention processing of social exclusion.

The temporal models of ostracism suggest that human beings be evolved an efficient pre-cognitive warning system to immediately detect and respond to ostracism (Zadro et al., 2004; Williams and Zadro, 2005; Williams, 2009; Williams and Nida, 2011). Reliably, the enhanced frontocentral P200 to averted gaze in our experiment reflect an earlier affective processing of social exclusion. Cautiously, the frontocentral N200 may reflect the intrinsic effect of “negative bias” in rapid automatic attention processing of social exclusion. Accordingly, we proposed that the frontocentral P200/N200 complex may be related to the early pre-cognitive warning system in the temporal models of ostracism.

In addition to the earlier P200 and N200 linked to affective and automatic attention processing of social exclusion, we also found that the LPP over the centroparietal areas in the time window of 370–700 ms was more positive-going for the averted gaze than for the direct gaze. In general, the centroparietal LPPs or P3b are associated with the high-level allocation of attentional resources for stimuli evaluation and categorization, and subsequent memory processing (Polich, 2007; Olofsson et al., 2008; Muñoz and Martín-Loeches, 2015). Specifically, some ERP studies on social exclusion, by using Cyberball task, have demonstrated that in relative to the included events, the excluded events can easily attract individuals’ attention due to their salience. Such effect can be indexed by greater P300 or LPP responses over the central-parietal regions (Crowley et al., 2010; Gutz et al., 2011). Furthermore, because of the positive correlation between the change in subjective rating of perceived ostracism intensity and the parietal P3b amplitude of difference waves (i.e., averted gaze minus direct gaze), the authors indicated that the P3b may index the perceived ostracism intensity which is regarded as cognitive stimulus evaluation and categorization of exclusion and inclusion (Gutz et al., 2011). Fortunately, in our study, for the averted condition, we found obviously positive correlation between the subjective rating of perceived ostracism intensity and the parietal LPP amplitude. Therefore, we suggest that the parietal LPP may have the similar function of P3b observed in Gutz et al.’s (2011) study.

Until now, a large literature has confirmed social function of the direction of eye gaze in social and non-verbal communication. For instance, several studies have suggested that, in the context of neutral facial expression, the averted gaze signal disinterest (Strick et al., 2008; Itier and Batty, 2009), and may be related to avoidance (Hietanen et al., 2008; Pönkänen et al., 2011). In terms of the neural dynamics for social function of eye gaze, some ERP research even proposes that neural activity earlier than 300 ms post-stimulus may index the processing of gaze change independent of social context (Conty et al., 2007; Itier and Batty, 2009; Nummenmaa and Calder, 2009), whereas, that the slow wave may after 300 ms index the processes associated with extracting social meaning (Carrick et al., 2007; Itier et al., 2007). To be noted, in Carrick et al.’s (2007) study, the P500 amplitude was greater for the gaze avoidance compared to direct gaze. Therefore, it is not surprising that the averted gaze elicited the stronger LPP response in our study. Here, the interviewers’ averted gaze may indicate that the interviewee cannot draw their attention and is less likely to be accepted by the club. Such gaze shift can be regarded as a kind of adverse social evaluation feedback containing obvious social meanings, e.g., potential indifference or avoidance. Its salience may attract the interviewees’ more attention leading to the high-level cognitive processing of stimulus evaluation. Finally, in consideration of temporal need-threat model suggesting that the reflective stage of reaction to ostracism last for several seconds (Williams, 2009; Williams and Nida, 2011), we believe that the centroparietal LPP being more pronounced for the averted gaze than for the direct gaze may index the preliminary cognitive evaluation and categorization on excluded and included events, and even the unspecific attentional distribution on excluded events still within the reflexive stage, that leads into a more situation-specific appraisal response during the later reflective stage.

## Conclusion

By using the modified Eye-gaze paradigm, this study examined the psychological and neural temporal dynamics of social exclusion. The ERP data and the subjective rating data showed that the frontocentral P200 was positively correlated with negative mood of excluded events, whereas, the centroparietal LPP was positively correlated with the perceived ostracism intensity. Both the P200 and LPP were more positive-going for excluded events than for included events. These findings suggest that brain responses sensitive to social exclusion can be divided into the early affective processing stage, linking to the early pre-cognitive warning system; and the late higher-order processes stage, demanding attentional resources for elaborate stimulus evaluation and categorization generally irrespective of certain situation.

## Author Contributions

YL: conception and design. JZ: collection of data. XQ, YZ and YL: analysis and interpretation of data. YL: drafting the article. YL and SG: revising the article.

## Conflict of Interest Statement

The authors declare that the research was conducted in the absence of any commercial or financial relationships that could be construed as a potential conflict of interest.

## Footnotes

^1^We also conducted the multilevel mediation analysis to test whether ERP responses mediate the effect of exclusion on mood changes. The effect of exclusion on the P200 amplitude reached significance, *p* < 0.05; but the effect of the P200 on mood changes did not reach significance, *p* > 0.1. The effect of exclusion on the LPP amplitude reached significance, *p* < 0.05; but the effect of the LPP on perceived ostracism intensity did not reach significance, *p* > 0.1. Therefore, the serial effect/causality was not found.

## References

[B1] Bolling D. Z. Pitskel N. B. Deen B. Crowley M. J. McPartland J. C. Mayes L. C.. (2011). Dissociable brain mechanisms for processing social exclusion and rule violation. Neuroimage 54, 2462–2471. 10.1016/j.neuroimage.2010.10.049 20974272 PMC3006641

[B2] Carretié L. Hinojosa J. A. Martín-Loeches M. Mercado F. Tapia M. (2004). Automatic attention to emotional stimuli: neural correlates. Hum. Brain Mapp. 22, 290–299. 10.1002/hbm.20037 15202107 PMC6871850

[B3] Carretié L. Mercado F. Tapia M. Hinojosa J. A. (2001). Emotion, attention and the ‘negativity bias’, studied through event-related potentials. Int. J. Psychophysiol. 41, 75–85. 10.1016/s0167-8760(00)00195-1 11239699

[B4] Carrick O. K. Thompson J. C. Epling J. A. Puce A. (2007). It’s all in the eyes: neural responses to socially significant gaze shifts. Neuroreport 18, 763–766. 10.1097/wnr.0b013e3280ebb44b 17471062 PMC2794043

[B5] Conty L. N’Diaye K. Tijus C. George N. (2007). When eye creates the contact! ERP evidence for early dissociation between direct and averted gaze motion processing. Neuropsychologia 45, 3024–3037. 10.1016/j.neuropsychologia.2007.05.017 17644145

[B6] Crowley M. J. Wu J. McCarty E. R. David D. H. Bailey C. A. Mayes L. C. (2009). Exclusion and micro-rejection: event-related potential response predicts mitigated distress. Neuroreport 20, 1518–1522. 10.1097/wnr.0b013e328330377a 19829163 PMC4457507

[B7] Crowley M. J. Wu J. Molfese P. J. Mayes L. C. (2010). Social exclusion in middle childhood: rejection events, slow-wave neural activity, and ostracism distress. Soc. Neurosci. 5, 483–495. 10.1080/17470919.2010.500169 20628967 PMC2991408

[B8] Deater-Deckard K. (2001). Annotation: recent research examining the role of peer relationships in the development of psychopathology. J. Child Psychol. Psychiatry 42, 565–579. 10.1111/1469-7610.00753 11464962

[B9] Delplanque S. Lavoie M. E. Hot P. Silvert L. Sequeira H. (2004). Modulation of cognitive processing by emotional valence studied through event-related potentials in humans. Neurosci. Lett. 356, 1–4. 10.1016/j.neulet.2003.10.014 14746887

[B10] Delplanque S. Silvert L. Hot P. Sequeira H. (2005). Event-related P3a and P3b in response to unpredictable emotional stimuli. Biol. Psychol. 68, 107–120. 10.1016/j.biopsycho.2004.04.006 15450691

[B11] DeWall C. N. MacDonald G. Webster G. D. Masten C. L. Baumeister R. F. Powell C.. (2010). Acetaminophen reduces social pain behavioral and neural evidence. Psychol. Sci. 21, 931–937. 10.1177/0956797610374741 20548058

[B12] DeWall C. N. Masten C. L. Powell C. Combs D. Schurtz D. R. Eisenberger N. I. (2012). Do neural responses to rejection depend on attachment style? An fMRI study. Soc. Cogn. Affect. Neurosci. 7, 184–192. 10.1093/scan/nsq107 21467049 PMC3277372

[B13] Eisenberger N. I. Inagaki T. K. Muscatell K. A. Haltom K. E. B. Leary M. R. (2011). The neural sociometer: brain mechanisms underlying state self-esteem. J. Cogn. Neurosci. 23, 3448–3455. 10.1162/jocn_a_00027 21452934

[B14] Eisenberger N. I. Lieberman M. D. Williams K. D. (2003). Does rejection hurt? An fMRI study of social exclusion. Science 302, 290–292. 10.1126/science.1089134 14551436

[B15] Feng C. Li W. Tian T. Luo Y. Gu R. Zhou C.. (2014). Arousal modulates valence effects on both early and late stages of affective picture processing in a passive viewing task. Soc. Neurosci. 9, 364–377. 10.1080/17470919.2014.896827 24601745

[B16] Gutz L. Küpper C. Renneberg B. Niedeggen M. (2011). Processing social participation: an event-related brain potential study. Neuroreport 22, 453–458. 10.1097/wnr.0b013e3283476b67 21558970

[B17] Guyer A. E. Choate V. R. Pine D. S. Nelson E. E. (2012). Neural circuitry underlying affective response to peer feedback in adolescence. Soc. Cogn. Affect. Neurosci. 7, 81–92. 10.1093/scan/nsr043 21828112 PMC3252630

[B18] Guyer A. E. Lau J. Y. McClure-Tone E. B. Parrish J. Shiffrin N. D. Reynolds R. C.. (2008). Amygdala and ventrolateral prefrontal cortex function during anticipated peer evaluation in pediatric social anxiety. Arch. Gen. Psychiatry 65, 1303–1312. 10.1001/archpsyc.65.11.1303 18981342 PMC2717208

[B19] Guyer A. E. McClure-Tone E. B. Shiffrin N. D. Pine D. S. Nelson E. E. (2009). Probing the neural correlates of anticipated peer evaluation in adolescence. Child Dev. 80, 1000–1015. 10.1111/j.1467-8624.2009.01313.x 19630890 PMC2791675

[B20] Hietanen J. K. Leppänen J. M. Peltola M. J. Linna-Aho K. Ruuhiala H. J. (2008). Seeing direct and averted gaze activates the approach-avoidance motivational brain systems. Neuropsychologia 46, 2423–2430. 10.1016/j.neuropsychologia.2008.02.029 18402988

[B21] Huang Y. Luo Y. (2006). Temporal course of emotional negativity bias: an ERP study. Neurosci. Lett. 398, 91–96. 10.1016/j.neulet.2005.12.074 16446031

[B23] Itier R. J. Alain C. Kovacevic N. McIntosh A. R. (2007). Explicit versus implicit gaze processing assessed by ERPs. Brain Res. 1177, 79–89. 10.1016/j.brainres.2007.07.094 17916340

[B22] Itier R. J. Batty M. (2009). Neural bases of eye and gaze processing: the core of social cognition. Neurosci. Biobehav. Rev. 33, 843–863. 10.1016/j.neubiorev.2009.02.004 19428496 PMC3925117

[B24] Kaunhoven R. J. Dorjee D. (2017). How does mindfulness modulate self-regulation in pre-adolescent children? An integrative neurocognitive review. Neurosci. Biobehav. Rev. 74, 163–184. 10.1016/j.neubiorev.2017.01.007 28108415

[B25] Krill A. Platek S. M. (2009). In-group and out-group membership mediates anterior cingulate activation to social exclusion. Front. Evol. Neurosci. 1:1. 10.3389/neuro.18.001.2009 19597546 PMC2704010

[B26] Kross E. Egner T. Ochsner K. Hirsch J. Downey G. (2007). Neural dynamics of rejection sensitivity. J. Cogn. Neurosci. 19, 945–956. 10.1162/jocn.2007.19.6.945 17536965

[B27] Ladd G. W. (2006). Peer rejection, aggressive or withdrawn behavior, and psychological maladjustment from ages 5 to 12: an examination of four predictive models. Child Dev. 77, 822–846. 10.1111/j.1467-8624.2006.00905.x 16942492

[B28] Masten C. L. Eisenberger N. I. Borofsky L. A. Pfeifer J. H. McNealy K. Mazziotta J. C.. (2009). Neural correlates of social exclusion during adolescence: understanding the distress of peer rejection. Soc. Cogn. Affect. Neurosci. 4, 143–157. 10.1093/scan/nsp007 19470528 PMC2686232

[B29] Masten C. L. Telzer E. H. Fuligni A. J. Lieberman M. D. Eisenberger N. I. (2012). Time spent with friends in adolescence relates to less neural sensitivity to later peer rejection. Soc. Cogn. Affect. Neurosci. 7, 106–114. 10.1093/scan/nsq098 21183457 PMC3252626

[B30] Moor B. G. Güroğlu B. Op de Macks Z. A. Rombouts S. A. Van der Molen M. W. Crone E. A. (2012). Social exclusion and punishment of excluders: neural correlates and developmental trajectories. Neuroimage 59, 708–717. 10.1016/j.neuroimage.2011.07.028 21791248

[B31] Muñoz F. Martín-Loeches M. (2015). Electrophysiological brain dynamics during the esthetic judgment of human bodies and faces. Brain Res. 1594, 154–164. 10.1016/j.brainres.2014.10.061 25451119

[B32] Nummenmaa L. Calder A. J. (2009). Neural mechanisms of social attention. Trends Cogn. Sci. 13, 135–143. 10.1016/j.tics.2008.12.006 19223221

[B33] Olofsson J. K. Nordin S. Sequeira H. Polich J. (2008). Affective picture processing: an integrative review of ERP findings. Biol. Psychol. 77, 247–265. 10.1016/j.biopsycho.2007.11.006 18164800 PMC2443061

[B34] Onoda K. Okamoto Y. Nakashima K. I. Nittono H. Ura M. Yamawaki S. (2009). Decreased ventral anterior cingulate cortex activity is associated with reduced social pain during emotional support. Soc. Neurosci. 4, 443–454. 10.1080/17470910902955884 19562631

[B35] Polich J. (2007). Updating P300: an integrative theory of P3a and P3b. Clin. Neurophysiol. 118, 2128–2148. 10.1016/j.clinph.2007.04.019 17573239 PMC2715154

[B36] Pönkänen L. M. Peltola M. J. Hietanen J. K. (2011). The observer observed: frontal EEG asymmetry and autonomic responses differentiate between another person’s direct and averted gaze when the face is seen live. Int. J. Psychophysiol. 82, 180–187. 10.1016/j.ijpsycho.2011.08.006 21893108

[B37] Ponz A. Montant M. Liegeois-Chauvel C. Silva C. Braun M. Jacobs A. M.. (2014). Emotion processing in words: a test of the neural re-use hypothesis using surface and intracranial EEG. Soc. Cogn. Affect. Neurosci. 9, 619–627. 10.1093/scan/nst034 23482627 PMC4014107

[B38] Schmitz J. Scheel C. N. Rigon A. Gross J. J. Blechert J. (2012). You don’t like me, do you? Enhanced ERP responses to averted eye gaze in social anxiety. Biol. Psychol. 91, 263–269. 10.1016/j.biopsycho.2012.07.004 22820039

[B39] Schutter D. J. de Haan E. H. van Honk J. (2004). Functionally dissociated aspects in anterior and posterior electrocortical processing of facial threat. Int. J. Psychophysiol. 53, 29–36. 10.1016/j.ijpsycho.2004.01.003 15172133

[B40] Semlitsch H. V. Anderer P. Schuster P. Presslich O. (1986). A solution for reliable and valid reduction of ocular artifacts, applied to the P300 ERP. Psychophysiology 23, 695–703. 10.1111/j.1469-8986.1986.tb00696.x 3823345

[B41] Silk J. S. Stroud L. R. Siegle G. J. Dahl R. E. Lee K. H. Nelson E. E. (2012). Peer acceptance and rejection through the eyes of youth: pupillary, eyetracking and ecological data from the Chatroom Interact task. Soc. Cogn. Affect. Neurosci. 7, 93–105. 10.1093/scan/nsr044 21775386 PMC3252631

[B42] Strick M. Holland R. W. van Knippenberg A. (2008). Seductive eyes: attractiveness and direct gaze increase desire for associated objects. Cognition 106, 1487–1496. 10.1016/j.cognition.2007.05.008 17601526

[B43] Thierry G. Roberts M. V. (2007). Event-related potential study of attention capture by affective sounds. Neuroreport 18, 245–248. 10.1097/WNR.0b013e328011dc95 17314665

[B44] Twenge J. M. Baumeister R. F. DeWall C. N. Ciarocco N. J. Bartels J. M. (2007). Social exclusion decreases prosocial behavior. J. Pers. Soc. Psychol. 92, 56–66. 10.1037/0022-3514.92.1.56 17201542

[B45] Twenge J. M. Baumeister R. F. Tice D. M. Stucke T. S. (2001). If you can’t join them, beat them: effects of social exclusion on aggressive behavior. J. Pers. Soc. Psychol. 81, 1058–1069. 10.1037/0022-3514.81.6.1058 11761307

[B46] van der Veen F. M. van der Molen M. W. Sahibdin P. P. Franken I. H. (2014). The heart-break of social rejection versus the brain wave of social acceptance. Soc. Cogn. Affect. Neurosci. 9, 1346–1351. 10.1093/scan/nst120 23887821 PMC4158370

[B47] Wang X. Huang Y. Ma Q. Li N. (2012). Event-related potential P2 correlates of implicit aesthetic experience. Neuroreport 23, 862–866. 10.1097/WNR.0b013e3283587161 22922601

[B48] Williams K. D. (1997). “Social ostracism,” in Aversive Interpersonal Behaviors. The Springer Series in Social/Clinical Psychology, ed. Kowalski R. M. (Boston, MA: Springer), 133–170.

[B49] Williams K. D. (2009). Ostracism: a temporal need-threat model. Adv. Exp. Soc. Psychol. 41, 275–314. 10.1016/S0065-2601(08)00406-1

[B50] Williams K. D. Nida S. A. (2011). Ostracism: consequences and coping. Curr. Dir. Psychol. Sci. 20, 71–75. 10.1177/0963721411402480

[B52] Williams K. D. Shore W. J. Grahe J. E. (1998). The silent treatment: perceptions of its behaviors and associated feelings. Group Process. Intergroup Relat. 1, 117–141. 10.1177/1368430298012002

[B51] Williams K. D. Zadro L. (2005). “Ostracism: the indiscriminate early detection system,” in The Social Outcast: Ostracism, Social Exclusion, Rejection, and Bullying, eds Williams K. D. Forgas J. P. Hippel W. V. (New York, NY: Psychology Press), 19–34.

[B53] Wirth J. H. Sacco D. F. Hugenberg K. Williams K. D. (2010). Eye gaze as relational evaluation: averted eye gaze leads to feelings of ostracism and relational devaluation. Pers. Soc. Psychol. Bull. 36, 869–882. 10.1177/0146167210370032 20505162

[B54] Zadro L. Williams K. D. Richardson R. (2004). How low can you go? Ostracism by a computer is sufficient to lower self-reported levels of belonging, control, self-esteem, and meaningful existence. J. Exp. Soc. Psychol. 40, 560–567. 10.1016/j.jesp.2003.11.006

[B55] Zhu C. He W. Qi Z. Wang L. Song D. Zhan L.. (2015). The time course of emotional picture processing: an event-related potential study using a rapid serial visual presentation paradigm. Front. Psychol. 6:954. 10.3389/fpsyg.2015.00954 26217276 PMC4497308

